# Recombinant Thrombomodulin Domain 1 Promotes Diabetic Corneal Wound Healing by Inhibiting HMGB1 Production and NLRP3 Inflammasome

**DOI:** 10.1155/mi/8089754

**Published:** 2026-01-06

**Authors:** Kuan-Ying Chen, I-Chen Peng, Hua-Lin Wu, Cheng-Hsiang Kuo, Yi-Hsun Huang

**Affiliations:** ^1^ Education Center, National Cheng Kung University Hospital, College of Medicine, National Cheng Kung University, Tainan, Taiwan, ncku.edu.tw; ^2^ Department of Ophthalmology, National Cheng Kung University Hospital, College of Medicine, National Cheng Kung University, 138 Sheng-Li Rd., Tainan, 704, Taiwan, ncku.edu.tw; ^3^ Department of Biochemistry and Molecular Biology, College of Medicine, National Cheng Kung University, Tainan, Taiwan, ncku.edu.tw; ^4^ Department of Physiology, College of Medicine, National Cheng Kung University, Tainan, Taiwan, ncku.edu.tw; ^5^ International Center for Wound Repair and Regeneration, National Cheng Kung University, Tainan, Taiwan, ncku.edu.tw

## Abstract

**Purpose:**

To investigate the therapeutic potential of recombinant thrombomodulin domain 1 (rTMD1) in diabetic corneal wound healing and to elucidate its underlying mechanisms using in vitro and in vivo models.

**Methods:**

rTMD1 was produced using the *Pichia pastoris* expression system and purified. Human corneal epithelial cells (HCECs) were cultured under normal glucose (NG) and high glucose (HG) conditions, with or without rTMD1 treatment. Wound healing rates were evaluated using a scratch assay. Diabetes was induced in C57BL/6 mice via streptozotocin (STZ) injections. Corneal wounds were created and treated with rTMD1 or PBS, and wound healing was assessed via fluorescein staining. Inflammatory markers, including HMGB1, TLR4, NLRP3, and IL‐1β, were analyzed via quantitative PCR (qPCR), Western blot, and immunofluorescence staining.

**Results:**

In vitro, HCECs treated with rTMD1 under HG conditions demonstrated a higher wound healing rate compared to untreated cells (*p* = 0.0049). In vivo, rTMD1 significantly enhanced corneal wound healing in diabetic mice, with faster wound closure compared to PBS‐treated controls at 24 h (*p* = 0.005) and 48 h (*p* < 0.0001). rTMD1 treatment reduced the expression of HMGB1, TLR4, NLRP3, and IL‐1β at both mRNA and protein levels, indicating suppression of inflammation.

**Conclusions:**

Topical application of rTMD1 promotes corneal epithelial wound healing in diabetic conditions by inhibiting HMGB1/TLR4/NLRP3‐mediated inflammation. rTMD1 holds promise as a potential therapeutic agent for diabetic keratopathy, although further studies are needed to validate its clinical efficacy and safety.

## 1. Introduction

Diabetes mellitus (DM) is a prevalent and chronic metabolic disorder characterized by prolonged hyperglycemia and a range of complications, affecting ~529 million individuals worldwide in 2021 [1]. Diabetic keratopathy (DK) is one of the most common ocular complications of diabetes, with a reported prevalence of 47%–64% among diabetic patients [2, 3]. DK manifests clinically as persistent corneal erosion and ulceration, often accompanied by reduced corneal sensitivity, primarily due to impaired corneal wound healing and delayed nerve regeneration [3]. If left untreated or poorly managed, DK can progress to corneal perforation and irreversible vision loss. Current management strategies focus on controlling blood glucose levels and providing symptomatic relief through topical antibiotics and lubricants [3]. However, these approaches have limited efficacy, as they do not address the underlying issue of delayed corneal wound healing associated with diabetes. Developing treatments that specifically target the mechanisms responsible for impaired healing in the diabetic cornea may offer better therapeutic outcomes and improve prognosis.

Recent studies suggest that excessive inflammation and sensory neuropathy are key contributors to DK [4]. Proper corneal wound healing requires a delicate balance between inflammation and regeneration. However, in DK, hyperglycemia‐induced accumulation of advanced glycation end‐products (AGEs) and reactive oxygen species (ROS) disrupt this balance by impairing cellular proliferation, exacerbating inflammation, and increasing cell death, ultimately leading to delayed wound healing [5]. Moreover, recent findings indicate that AGEs and ROS can activate the Nod‐like receptor protein 3 (NLRP3) inflammasome pathway, resulting in elevated production of interleukin‐1β (IL‐1β) and interleukin‐18 (IL‐18). This cascade promotes pyroptosis, further contributing to impaired corneal wound healing [6].

Thrombomodulin (TM) is a multifunctional transmembrane glycoprotein composed of five distinct domains: a lectin‐like domain (D1), a domain containing six epidermal growth factor (EGF)‐like structures (D2), a serine‐ and threonine‐rich domain (D3), a transmembrane domain (D4), and a cytoplasmic domain (D5) [7]. Initially identified as a thrombin cofactor with anticoagulant properties in vascular endothelial cells, TM is now increasingly recognized for its anti‐inflammatory functions, particularly those attributed to its lectin‐like domain (TMD1) [8, 9]. Studies have shown that TM can sequester high‐mobility group box 1 (HMGB1), a danger‐associated molecular pattern (DAMP), thereby mitigating the inflammatory response [10]. Furthermore, research has demonstrated that TMD1 can alleviate diabetic nephropathy in mice by inhibiting NF‐κB/NLRP3 inflammasome‐mediated inflammation and apoptosis [11]. In the cornea, our previous studies have shown that TM expression increases during the early stages of wound healing and gradually declines upon wound closure. Additionally, we have found that the recombinant TM EGF‐like domain plus serine/threonine‐rich domain (rTMD23) can effectively promote corneal epithelial wound healing [12].

Given its anti‐inflammatory properties and ability to promote cutaneous wound healing, TM—particularly its lectin‐like domain (TMD1)—holds potential as a therapeutic agent for DK. However, the effects of TMD1 on diabetic corneal wound healing, as well as its underlying mechanisms, remain unclear. Therefore, this study aimed to explore the therapeutic potential of recombinant TMD1 (rTMD1) in diabetic corneal wound healing using primary human corneal epithelial cells (HCECs) and animal models.

## 2. Materials and Methods

### 2.1. Preparation of rTMD1 Protein

rTMD1 protein was produced using the Pichia pastoris expression system and subsequently purified through nickel‐chelating Sepharose columns (Amersham Pharmacia Biotech, Uppsala, Sweden), following previously established protocols [13]. The purity of the obtained rTMD1 protein was assessed by sodium dodecyl sulfate‐polyacrylamide gel electrophoresis (SDS‐PAGE).

### 2.2. DM Animal Model

Male C57BL/6 mice aged 7–9 weeks were used in this study. All animal experiments were conducted in accordance with the ARVO Statement for the Use of Animals in Ophthalmic and Vision Research, and the study protocol was approved by the Institutional Animal Care and Use Committee of National Cheng Kung University (IACUC Number 113016). Type 1 diabetes was induced by administering intraperitoneal injections of streptozotocin (STZ) at a dose of 50 mg/kg for five consecutive days [14]. 2 weeks after the final STZ injection, serum glucose levels were measured using a glucometer. Mice with serum glucose levels exceeding 250 mg/dL were classified as diabetic.

### 2.3. In Vivo Corneal Epithelial Wound Healing and Treatment

Prior to the procedure, both normal and diabetic mice were anesthetized via intraperitoneal injection of Zoletil (50 mg/kg; Sigma) combined with Xylazine (2.5 mg/kg; Sigma). Additionally, a drop of proparacaine hydrochloride 0.5% (Alcon) was applied to the cornea for local anesthesia. To create a standardized wound, a 2‐mm biopsy punch was used to demarcate the central corneal area of one eye, followed by gentle removal of the central corneal epithelium using an Algerbrush II corneal rust ring remover (Alloy Medical, San Mateo, CA). Topical treatment was administered twice: immediately after debridement (time 0) and again at 24 h postinjury. For the diabetic treatment group (DM‐rTMD1), rTMD1 in PBS at 2 mg/mL was instilled 5 μL per dose (10 μg/dose; total 20 μg/eye). Control groups (normal‐PBS and DM‐PBS) received PBS 5 μL at the same two time points. Corneal wound healing was monitored using fluorescein staining, and residual epithelial defects were photographed at 0, 24, and 48 h using a slit lamp microscope. Wound healing areas were quantified using ImageJ software to determine the percentage of wound closure. After 48 h, mice were euthanized by gradual‐fill carbon dioxide (CO_2_) inhalation at a flow rate of 30% chamber volume/min, in accordance with AVMA guidelines. Corneal specimens were then harvested for immunofluorescence staining.

### 2.4. Cell Cultures

Human primary corneal epithelial cells (HCECs) were purchased from cell biologics and cultured in human epithelial cell medium supplemented with EGF, hydrocortisone, and fetal bovine serum (FBS). To establish normal and high glucose (HG) conditions, the cells were seeded into six‐well plates and maintained in Dulbecco’s Modified Eagle Medium (DMEM; Thermo Fisher), containing either 5 mM glucose (normal glucose, NG) or 30 mM glucose (HG) for 48 h. Once the cells reached confluence, a scratch was created using a 200 µL pipette tip. The cells were then incubated in NG medium, HG medium, or HG medium supplemented with 20 nM TMD1 for 24 h. Images were captured at 0 and 24 h postscratch, and the percentage of wound closure was analyzed using ImageJ software.

### 2.5. Real‐Time Quantitative PCR (qPCR)

Total RNA was extracted from HCECs using the Real Genomics Total RNA Extraction Kit (RBC Bioscience, Taiwan). qPCR was performed using PowerUp SYBR Green Master Mix (Thermo Fisher). The primers used in this study are listed in Table 1.

**Table 1:** List of primers.

Genes	Forward primer	Reverse primer
Hu HMGB1	GCG AAG AAA CTG GGA GAG ATG TG	GCA TCA GGC TTT CCT TTA GCT CG
Hu TLR4	CCC TGA GGC ATT TAG GCA GCT A	AGG TAG AGA GGT GGC TTA GGC T
Hu NLRP3	CCA TCG GCA AGA CCA AGA	ACA GGC TCA GAA TGC TCA TC
Hu IL‐1β	CAG CCA ATC TTC ATT GCT CA	AGT CAT CCT CAT TGC CAC TGT
Hu GAPDH	TGA CTT CAA CAG CGA CAC CCA	CAC CCT GTT GCT GTA GCC AAA
Ms HMGB1	TGG GCG ACT CTG TGC CTC	GCC TCT CGG CTT TTT AGG ATC
Ms TLR4	AGA AAT TCC TGC AGT GGG TCA	TCT CTA CAG GTG TTG CAC ATG TCA
Ms NLRP3	CTG CGG ACT GTC CCA TCA AT	AGG TTG CAG AGC AGG TGC TT
Ms IL‐1β	TGT AAT GAA AGA CGG CAC ACC	TCT TCT TTG GGT ATT GCT TGG
Ms GAPDH	CTC CAT TCC TCC TCC AGA CAC T	GCC TTC ATG TCT ATA GGT GAT GC

Abbreviations: Hu, human; Ms, mouse.

### 2.6. Western Blot Analysis

Total protein was extracted from HCECs using radioimmunoprecipitation assay (RIPA) buffer. Approximately 20 μg of total protein was separated on a 10% SDS‐PAGE gel and subsequently transferred onto a polyvinylidene difluoride (PVDF) membrane. The membranes were blocked with 1% bovine serum albumin (BSA) at room temperature for 1 h, followed by overnight incubation at 4°C with primary antibodies targeting HMGB1 (ab18256, Abcam), TLR4 (AF7017, Affinity), NLRP3 (DF15549, Affinity), IL‐1β (ab315084, Abcam), and GAPDH (sc‐32233, Santa Cruz). After incubation, species‐specific secondary antibodies were applied at room temperature for 1 h. Protein signals were visualized using an enhanced chemiluminescence (ECL) reagent and captured with an Amersham Imager 600 (GE Healthcare, USA). Band intensities were quantified using ImageJ software.

### 2.7. Immunofluorescence Staining

Corneal samples were collected, fixed, and embedded in paraffin, then sectioned at a thickness of 5 μm. Following deparaffinization, rehydration, and antigen retrieval, the sections were permeabilized with 0.1% Triton X‐100 and blocked with 5% goat serum for 1 h at room temperature. The sections were then incubated overnight at 4°C with primary antibodies against HMGB1 (1:100, ab18256, Abcam), TLR4 (1:100, AF7017, Affinity), NLRP3 (1:100, DF15549, Affinity), IL‐1β (1:100, GTX74034, GeneTex), F4/80 (1:100, ab6640, Abcam), and NIMP‐R14 (1:100, ab2557, Abcam). After washing, the sections were incubated for 1 h at room temperature with Alexa Fluor 488– or 546–conjugated anti‐rabbit IgG (Merck), and nuclei were counterstained with DAPI (Hoechst 33342, Merck). Negative controls were processed in parallel by omitting primary antibodies and incubating them with secondary antibodies only under identical imaging settings (Figure S1).

For quantitative analysis, immunofluorescent images were captured under identical microscope settings across all groups. Three nonoverlapping high‐power fields (HPFs) per section were randomly selected. Both epithelial and stromal regions were jointly evaluated for quantification. For HMGB1, TLR4, NLRP3, and IL‐1β, mean fluorescence intensity (MFI) per HPF was measured using ImageJ by outlining the region of interest and subtracting background signal from negative control sections. For F4/80 and NIMP‐R14, positively stained cells were manually counted per HPF. All values were statistically compared between groups to assess relative expression levels.

### 2.8. Statistical Analysis

All data are expressed as the mean ± standard deviation (SD). An unpaired two‐tailed Student’s *t*‐test was used for comparisons between two groups, while one‐way ANOVA was applied for comparisons among three groups, with statistical significance set at *p* < 0.05. All statistical analyses were performed using Prism 8.0 software.

## 3. Results

### 3.1. rTMD1 Enhances HCECs Wound Healing Under Hyperglycemic Conditions

**Figure 1:**
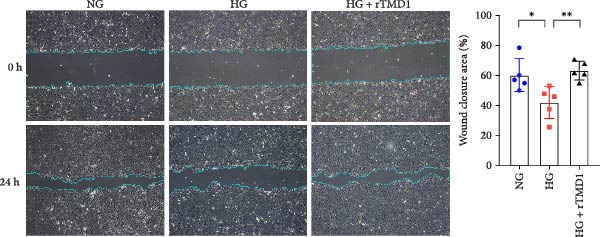
Recombinant thrombomodulin Domain 1 promoted human corneal epithelial cell wound closure under hyperglycemic environment. (NG: 5 mM glucose medium; HG: 30 mM glucose medium;  ^∗^ *p* < 0.05;  ^∗∗^ *p* < 0.01; *n* = 5 per group).

To determine whether rTMD1 promotes corneal wound healing under hyperglycemic conditions, we established a scratch injury model using primary HCECs. The cells were cultured in NG medium, HG medium, or HG medium supplemented with rTMD1 (20 nM) following injury induced by a pipette tip. At 24 h postwounding, the mean wound healing percentages were 60.25% in the NG group, 41.93% in the HG group, and 63.23% in the HG + rTMD1 group. The wound healing rate was significantly lower in the HG group compared to the NG group (*n* = 5; *p* = 0.0281), whereas the HG + rTMD1 group showed a significantly higher wound healing rate compared to the HG group (*n* = 5; *p* = 0.0049; Figure 1). These findings indicate that a high‐glucose environment impairs human corneal epithelial wound healing, while rTMD1 enhances the healing process under hyperglycemic conditions.

### 3.2. rTMD1 Suppresses HMGB1 Production and NLRP3 Inflammasome‐Mediated Inflammation in HCECs

**Figure 2:**
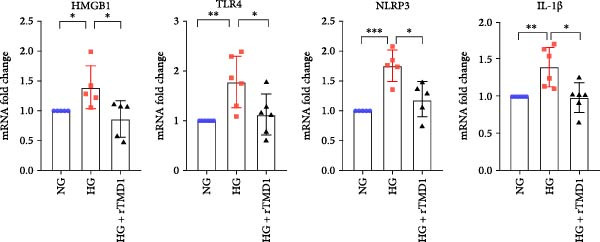
Recombinant thrombomodulin domain 1 inhibits the mRNA expression of the HMGB1/TLR4/NLRP3/IL‐1β signaling pathway in human corneal epithelial cells under a hyperglycemic condition after 24 h of treatment. (NG: 5 mM glucose medium; HG: 30 mM glucose medium;  ^∗^ *p* < 0.05;  ^∗∗^ *p* < 0.01;  ^∗∗∗^ *p* < 0.001; HMGB1: *n* = 5; TLR4: *n* = 6; NLRP3: *n* = 5; IL‐1β: *n* = 6).

Previous studies have demonstrated that TMD1 alleviates inflammation via the NLRP3 pathway in diabetic nephropathy [11]. To explore the potential mechanism by rTMD1 promotes corneal wound healing under diabetic conditions, we analyzed mRNA and total protein levels in HCECs 24 h after injury using qPCR and Western blot assays. The mRNA expression levels of HMGB1, TLR4, NLRP3, and IL‐1β were significantly higher in the HG group compared to the NG group (HMGB1: *p* = 0.038, *n* = 5; TLR4: *p* = 0.0043, *n* = 6; NLRP3: *p* = 0.0002, *n* = 5; IL‐1β: *p* = 0.0041, *n* = 6; Figure 2), indicating that HG conditions promote inflammation. However, treatment with rTMD1 significantly reduced the mRNA expression of these inflammatory markers in the HG + rTMD1 group compared to the HG group (HMGB1: *p* = 0.0363, *n* = 5; TLR4: *p* = 0.0372, *n* = 6; NLRP3: *p* = 0.0128, *n* = 5; IL‐1β: *p* = 0.0126, *n* = 6; Figure 2).

**Figure 3:**
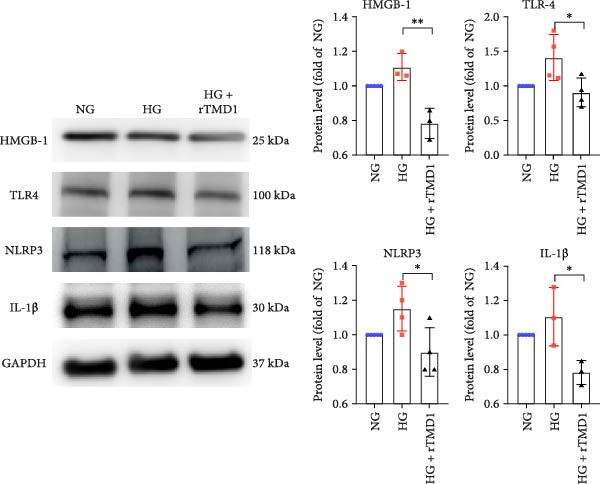
Recombinant thrombomodulin domain 1 inhibits the protein expression of the HMGB1/TLR4/NLRP3/IL‐1β signaling pathway in human corneal epithelial cells under a hyperglycemic condition after 24 h of treatment. (NG: 5 mM glucose medium; HG: 30 mM glucose medium;  ^∗^ *p* < 0.05;  ^∗∗^ *p* < 0.01; HMGB1: *n* = 3; TLR4: *n* = 4; NLRP3: *n* = 4; IL‐1β: *n* = 3).

Similarly, Western blot analysis showed that protein levels of HMGB1, TLR4, NLRP3, and IL‐1β were significantly lower in the HG + rTMD1 group compared to the HG group (HMGB1: *p* = 0.0083, *n* = 3; TLR4: *p* = 0.0418, *n* = 4; NLRP3: *p* = 0.0401, *n* = 4; IL‐1β: *p* = 0.0383, *n* = 3; Figure 3). These findings suggest that rTMD1 mitigates inflammation by suppressing HMGB1 production and inhibiting the TLR4/NLRP3/IL‐1β signaling pathway, which may contribute to improved corneal wound healing in diabetic conditions.

### 3.3. rTMD1 Promotes Corneal Wound Healing in STZ‐Induced Diabetic Mice

**Figure 4:**
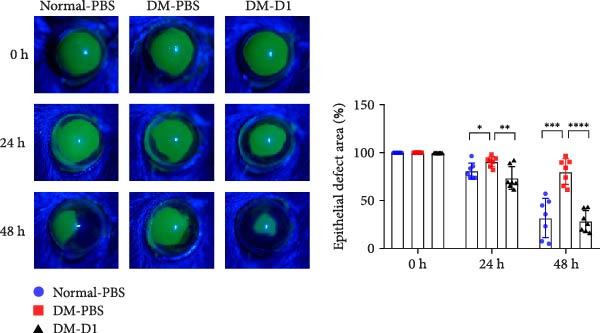
Recombinant thrombomodulin domain 1 promoted corneal epithelial wound healing in diabetic mice. (Normal‐PBS: normal mice treated with PBS, DM‐PBS: STZ‐induced diabetic mice treated with PBS, DM‐D1: STZ‐induced diabetic mice treated with rTMD1;  ^∗^ *p* < 0.05;  ^∗∗^ *p* < 0.01;  ^∗∗∗^ *p* < 0.001;  ^∗∗∗∗^ *p* < 0.0001; *n* = 7 per group).

To investigate the therapeutic potential of rTMD1 in diabetic corneal wound healing in vivo, we first induced type 1 diabetes in C57BL/6 mice via intraperitoneal injection of STZ. A total of 30 mice were used in this study, with 10 mice planned for each group. Among the 20 mice that received STZ for diabetes induction, six did not reach the hyperglycemia threshold and were excluded, resulting in 14 diabetic mice for analysis. A 2‐mm diameter wound was then created in the central cornea. Normal control mice received 5 μL of PBS, whereas diabetic mice received either 5 μL of PBS (DM‐PBS group) or rTMD1 (DM‐D1 group). Fluorescein staining showed that the DM‐PBS group had significantly larger residual corneal defects than the normal control group at both 24 h (*n* = 7; DM‐PBS: 90.8 ± 5.3% vs. normal‐PBS: 81.0 ± 8.4%; *p* = 0.0235) and 48 h (*n* = 7; DM‐PBS: 80.4 ± 13.6% vs. normal‐PBS: 32.0 ± 20.6%; *p* = 0.0002) after wounding (Figure 4). By contrast, the DM‐D1 group exhibited significantly smaller defect areas than the DM‐PBS group at 24 h (*n* = 7; 73.9 ± 12.0% vs. 90.8 ± 5.3%; *p* = 0.005) and 48 h (*n* = 7; 28.5 ± 11.3% vs. 80.4 ± 13.6%; *p* < 0.0001) (Figure 4; Figure S2). These findings indicate that rTMD1 promotes corneal epithelial wound healing in diabetic mice.

### 3.4. rTMD1 Reduces Leukocyte Infiltration, HMGB1 Expression, and Suppresses NLRP3 Inflammasome‐Related Inflammation in STZ‐Induced Diabetic Mice

**Figure 5:**
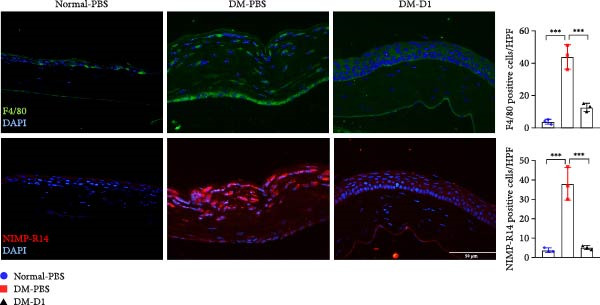
Immunofluorescence staining of F4/80 (macrophages) and NIMP‐R14 (neutrophils) in corneas from normal mice, diabetic mice treated with PBS, and diabetic mice treated with recombinant thrombomodulin domain 1. Bar graphs show the quantification of positive cells per high‐power field. (Normal‐PBS: normal mice treated with PBS, DM‐PBS: STZ‐induced diabetic mice treated with PBS, DM‐D1: STZ‐induced diabetic mice treated with rTMD1;  ^∗∗∗^ *p* < 0.001; *n* = 3 per group).

To further elucidate the anti‐inflammatory effects of rTMD1 in vivo, we examined leukocyte infiltration in the corneas of diabetic mice using immunofluorescence staining for F4/80 and NIMP‐R14. Compared to the normal‐PBS group, corneas from the DM‐PBS group exhibited markedly increased numbers of F4/80^+^ macrophages and NIMP‐R14^+^ neutrophils, indicating enhanced inflammatory cell infiltration under diabetic conditions. Notably, rTMD1 treatment significantly reduced the presence of both cell types in the DM‐D1 group (Figure 5), suggesting that rTMD1 attenuates inflammatory cell recruitment in diabetic corneas.

**Figure 6:**
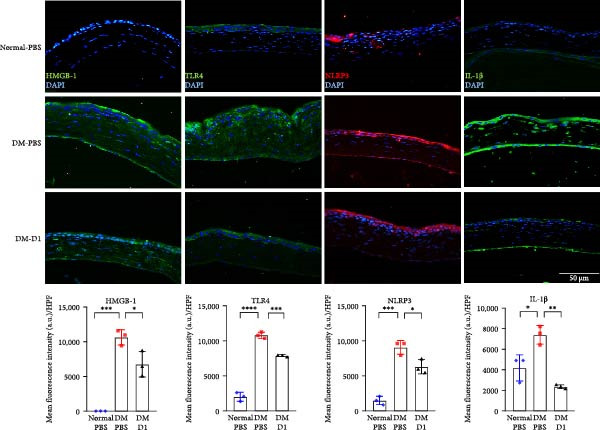
Immunofluorescence staining of HMGB1, TLR4, NLRP3, and IL‐1β in corneas from normal mice, diabetic mice treated with PBS, and diabetic mice treated with recombinant thrombomodulin domain 1. Bar graphs show the quantification of mean fluorescence intensity. (Normal‐PBS: normal mice treated with PBS, DM‐PBS: STZ‐induced diabetic mice treated with PBS, DM‐D1: STZ‐induced diabetic mice treated with rTMD1;  ^∗^ *p* < 0.05;  ^∗∗^ *p* < 0.01;  ^∗∗∗^ *p* < 0.001;  ^∗∗∗∗^ *p* < 0.0001; *n* = 3 per group).

In addition, we evaluated the expression of key inflammatory mediators in corneal sections from normal‐PBS, DM‐PBS, and DM‐D1 groups using immunofluorescence staining for HMGB1, TLR4, NLRP3, and IL‐1β. Diabetic corneas in the DM‐PBS group displayed elevated immunoreactivities for all four markers compared to normal controls. In contrast, rTMD1‐treated diabetic corneas exhibited markedly reduced fluorescence intensity for HMGB1, TLR4, NLRP3, and IL‐1β (Figure 6). These findings suggest that rTMD1 promotes diabetic corneal wound healing by suppressing leukocyte infiltration and downregulating HMGB1/TLR4/NLRP3/IL‐1β–mediated inflammation.

## 4. Discussion

DK is a common ocular complication of diabetes that affects the cornea, leading to delayed wound healing and impaired innervation. Although increasing evidence suggests that excessive inflammation and neuropathy induced by hyperglycemia are key contributors to DK, its complete pathogenesis and effective treatment options remain unclear. In this study, we demonstrated that rTMD1 inhibits HMGB1/TLR4/NLRP3‐mediated inflammation and accelerates corneal wound healing in both in vitro and in vivo diabetic models. These findings suggest that rTMD1 holds promise as a potential therapeutic option for DK.

TMD1, the lectin‐like domain of TM, has demonstrated both anti‐inflammatory and anti‐angiogenic properties, which have been beneficial in conditions such as diabetic nephropathy and oxygen‐induced retinopathy [11, 13]. These characteristics make TMD1 particularly well‐suited for treating DK. Therefore, in this study, we established in vitro and in vivo models to investigate the therapeutic potential and mechanisms of rTMD1 in diabetic corneal wound healing. In our HCECs scratch injury model, an HG environment significantly impaired wound healing, whereas treatment with rTMD1 effectively improved the healing rate. Similarly, in STZ‐induced diabetic mice, those treated with topical rTMD1 exhibited significantly faster wound closure compared to the PBS‐treated group. Importantly, no corneal neovascularization was observed following rTMD1 treatment, further supporting its suitability for corneal therapy. These results collectively indicate that rTMD1 effectively enhances corneal wound healing under diabetic conditions.

Previous studies have demonstrated that rTMD1 exerts anti‐inflammatory and tissue‐protective effects in a dose‐dependent manner. In models of angiogenesis, increasing concentrations of rTMD1 led to greater suppression of inflammatory signaling and pathological features [13]. For our study, a concentration of 20 nM was used in vitro, based on prior evidence of its efficacy in endothelial cells and macrophages. In vivo, we employed a topical dose of 2 mg/mL, which is consistent with previous studies using rTMD1 in corneal injury models [15]. Although we did not evaluate multiple doses, the selected concentrations are pharmacologically relevant and supported by earlier findings.

Molecular analyses using Western blot and qPCR in our in *vitro* model revealed that HG conditions significantly upregulated the expression of HMGB1, TLR4, NLRP3, and IL‐1β compared to NG controls. Treatment with rTMD1 markedly reduced the expression of these inflammatory markers. Consistently, immunofluorescence staining in our in vivo model confirmed that rTMD1 effectively suppressed HMGB1, TLR4, NLRP3, and IL‐1β expression, highlighting its anti‐inflammatory effects in diabetic corneas. In addition to suppressing inflammatory signaling, rTMD1 also reduced immune cell infiltration in diabetic corneas. Specifically, immunofluorescence analysis showed that rTMD1 significantly decreased the presence of F4/80^+^ macrophages and NIMP‐R14^+^ neutrophils, further supporting its role in mitigating corneal inflammation under hyperglycemic conditions.

HMGB1 is a typical DAMP that engages TLR4 to activate NF‐κB and prime the NLRP3 inflammasome, thereby amplifying IL‐1β–driven inflammation; diabetes‐related AGEs and ROS further potentiate NLRP3 activation and pyroptosis, delaying epithelial repair [6, 16]. In our models, rTMD1 reduced HMGB1, TLR4, NLRP3, and IL‐1β, and concomitantly lowered corneal F4/80^+^ macrophages and NIMP‐R14^+^ neutrophils, indicating broad dampening of the inflammatory milieu. By limiting this hyperinflammatory response, rTMD1 may promote a more favorable environment for inflammation resolution and tissue repair. Mechanistically, the lectin‐like domain of TM directly sequesters extracellular HMGB1, limiting its availability to stimulate pattern‐recognition receptors, and thereby attenuates downstream signaling [10]. In parallel, D1 neutralizes LPS by binding the Lewis‐Y determinant, which blunts TLR4‐dependent activation and reduces adhesion‐molecule and cytokine induction [7]. In diabetic tissues, D1 also enhances NRF2‐linked antioxidant defenses while constraining NF‐κB/NLRP3 signaling and apoptosis, offering a mechanistic bridge to our findings under high‐glucose stress [11]. Although we did not measure cleaved caspase‐1, cleaved or secreted IL‐1β, previous diabetic models have shown that D1 reduces caspase‐1 activity and lowers systemic IL‐1β and IL‐18, and under HG decreases cellular HMGB1 [11]. These findings are consistent with our inhibition of HMGB1, TLR4, NLRP3, and IL‐1β and support rTMD1 as a suppressor of inflammasome priming and activation.

Beyond extracellular sequestration, our data suggest that rTMD1 may also suppress HMGB1 production at the transcriptional level. In our model, rTMD1 treatment significantly reduced HMGB1 mRNA expression, indicating regulatory effects upstream of protein release. One possible explanation is that the reduction of corneal inflammation, as shown by decreased macrophage and neutrophil infiltration, leads to diminished HMGB1 synthesis and active secretion. Previous studies have shown that TMD1 attenuates NF‐κB activation, a key pathway controlling HMGB1 expression and release in inflammatory conditions [7, 17]. This feedback mechanism may contribute to the broader anti‐inflammatory and reparative actions of rTMD1 in diabetic corneal injury.

Evidence from nondiabetic corneal injury further supports a multi‐node action of rTMD1: in alkali‐burn models, it promotes epithelial recovery, shifts macrophage polarization from pro‐inflammatory M1 (CD86) to reparative M2 (CD206), and suppresses ERK/HIF‐1α with downstream reductions in VEGF and TNF‐α, changes that favor resolution and tissue repair [15]. Although a dedicated transmembrane receptor for rTMD1 has not been defined, the combination of HMGB1 sequestration and LPS neutralization, together with modulation of ERK/HIF‐1α–VEGF, NF‐κB, and NRF2 pathways, provides a coherent explanation for the broad attenuation of inflammatory signaling observed here [7, 11, 13]. With respect to proliferative effects, our study did not directly quantify epithelial proliferation; however, our previous study indicates that rTMD1 preserves cellular viability/proliferation while restraining pathogenic ERK/HIF‐1α activity, suggesting that accelerated closure in diabetes likely reflects both relief of inflammatory impediments and maintenance of reparative capacity rather than indiscriminate mitogenic stimulation [13]. Taken together, these data suggest that rTMD1 confers general anti‐inflammatory, pro‐repair benefits and, in the diabetic cornea, specifically corrects hyperglycemia‐sensitive HMGB1/TLR4/NLRP3 signaling to facilitate reepithelialization.

This study has several limitations relevant to translation. First, the in vitro HCEC assays and the STZ‐induced murine model do not fully recapitulate the chronic, multifactorial nature of human DK. Greater translational relevance will require evaluation in human diabetic corneal tissues and in larger diabetic animal models that more closely reflect human ocular anatomy, healing kinetics, and innervation. Second, although rTMD1 accelerated reepithelialization under hyperglycemic conditions, our endpoints primarily captured wound closure; studies that separately quantify epithelial proliferation and migration and that assess corneal innervation and sensory function are needed. Third, we employed a single concentration in vitro and a single topical regimen in vivo. Dose–response relationships, formulation optimization, and residence time at the corneal surface remain to be defined. Fourth, we did not perform dedicated ocular biosafety testing. Prior evidence supports rTMD1 tolerability: in an oxygen‐induced retinopathy model, rTMD1 did not induce cell toxicity and suppressed apoptosis, and separate corneal studies have applied topical rTMD1 at 2 mg/mL without reported safety signals [13, 15]. Nevertheless, a formal preclinical program for topical rTMD1 is still required, including standardized corneal toxicity and irritation assays. Finally, mechanistic analyses centered on the HMGB1/TLR4/NLRP3 axis. Additional pathways are likely to contribute, including ERK and HIF‐1 α regulation, macrophage polarization, and NRF2‐dependent antioxidant responses. Definitive causal assignment will require targeted perturbations and pathway‐level validation in diabetic corneal systems.

In conclusion, our study demonstrates that topical application of rTMD1 enhances corneal wound healing in diabetes by inhibiting HMGB1/TLR4/NLRP3/IL‐1β–mediated inflammation. These findings underscore the potential of rTMD1 as a promising therapeutic approach for managing DK. Further studies are needed to validate these findings and explore the broader therapeutic implications of rTMD1 in clinical settings.

## Conflicts of Interest

The authors declare no conflicts of interest.

## Author Contributions

**Kuan-Ying Chen:** methodology, investigation, writing – original draft. **I-Chen Peng:** methodology, investigation. **Hua-Lin Wu:** conceptualization, methodology. **Cheng-Hsiang Kuo:** methodology, writing – review and editing. **Yi-Hsun Huang:** conceptualization, writing – review and editing, project administration.

## Funding

This study was supported by the National Science and Technology Council, Taiwan (Grants 111‐2314‐B‐006‐072‐MY3 awarded to Yi‐Hsun Huang; 113‐2811‐B‐006‐003 and 113‐2326‐B‐006‐001‐MY3 awarded to Cheng‐Hsiang Kuo).

## Supporting Information

Additional supporting information can be found online in the Supporting Information section.

## Supporting information


**Supporting Information** Figure S1: Negative control for immunofluorescence staining. Figure S2: Additional representative fluorescein‐stained corneas supporting Figure 4.

## Data Availability

The data that support the findings of this study are available from the corresponding author upon reasonable request.
