# Supplementary figures and images for: Recombinant Thrombomodulin Domain 1 Promotes Diabetic Corneal Wound Healing by Inhibiting HMGB1 Production and NLRP3 Inflammasome
Source: Mediators Inflamm. 2026 Jan 6;2026:8089754. doi: 10.1155/mi/8089754 (PMC12771630; doi:10.1155/mi/8089754)

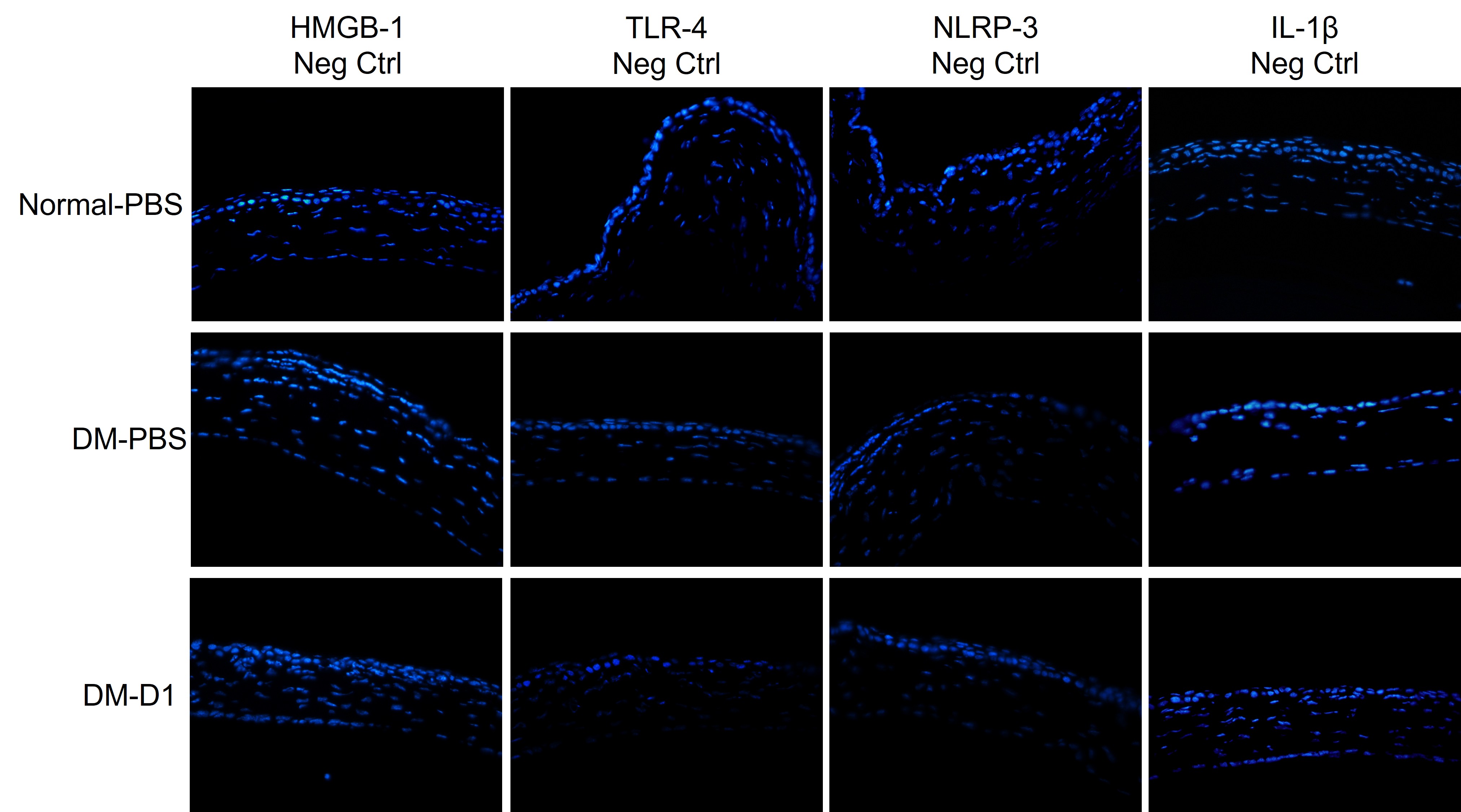

Supplement: Supplementary file 1 — Supporting Information Figure S1: Negative control for immunofluorescence staining. Figure S2: Additional representative fluorescein‐stained corneas supporting Figure 4. [file MI-2026-8089754-s001.zip › Supplementary figure 1.jpg]

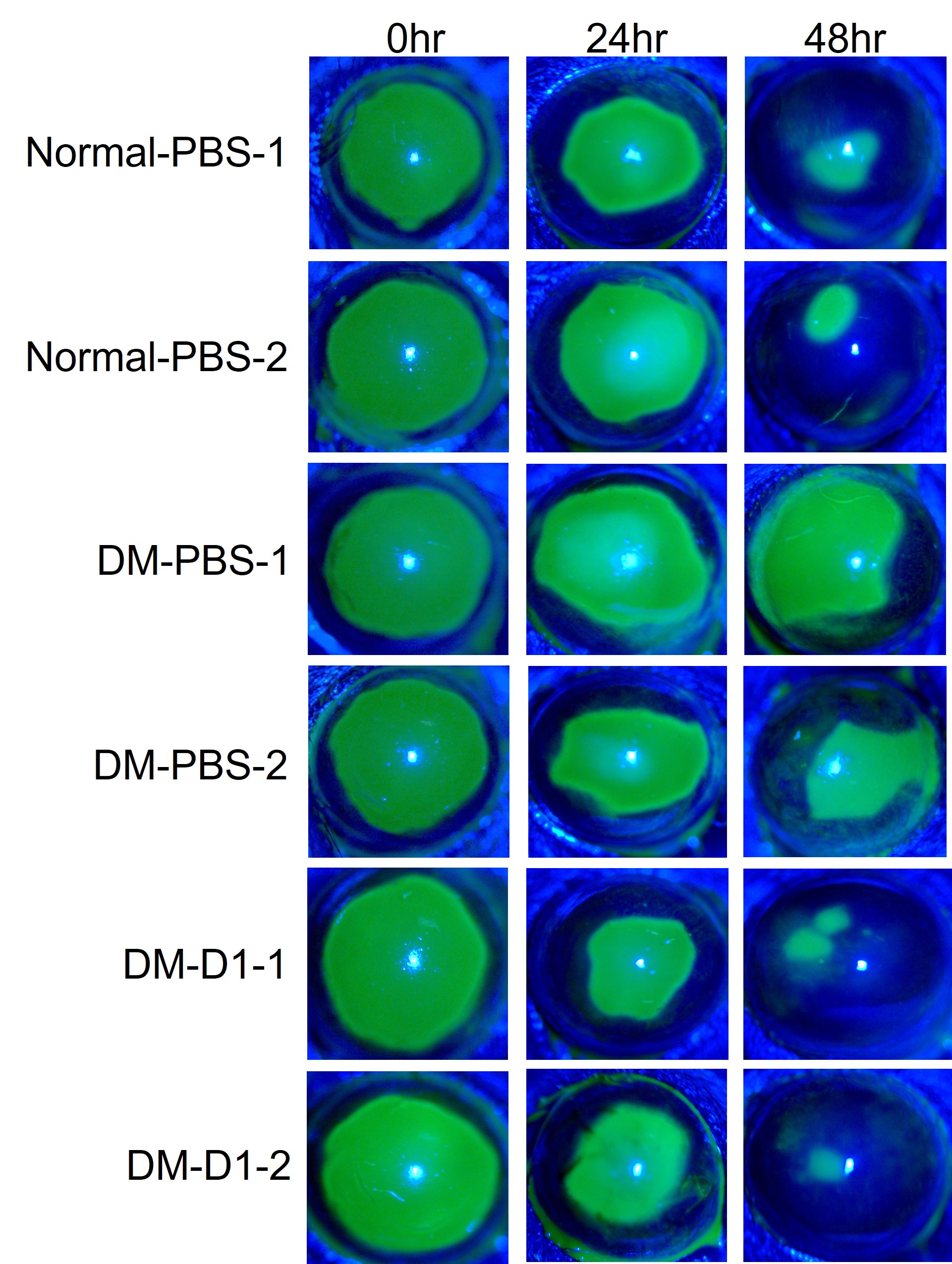

Supplement: Supplementary file 1 — Supporting Information Figure S1: Negative control for immunofluorescence staining. Figure S2: Additional representative fluorescein‐stained corneas supporting Figure 4. [file MI-2026-8089754-s001.zip › Supplementary figure 2.jpg]
